# Development of the dog executive function scale (DEFS) for adult dogs

**DOI:** 10.1007/s10071-022-01629-1

**Published:** 2022-05-17

**Authors:** Maike Foraita, Tiffani Howell, Pauleen Bennett

**Affiliations:** Anthrozoology Research Group, School of Psychology and Public Health, La Trobe University, Bendigo, Australia

**Keywords:** Behavioural regulation, Dog cognition, Dog behaviour, Working dogs

## Abstract

Executive functions (EFs) are cognitive processes that are used to effortfully self-regulate behaviour and might be important for dogs’ success in working and pet roles. Currently, studies are assessing dogs’ EF skills through often laborious cognitive measures, leading to small sample sizes and lacking measures of reliability. A complementary method is needed. The aim of this study was to develop a dog executive function scale (DEFS) for adult dogs. Focus groups were held with people working with dogs professionally to refine a pool of items describing dog behaviours related to EF. A survey was distributed online to a convenience sample of *N* = 714 owners of adult dogs. Exploratory and confirmatory factor analysis identified six distinguishable factors named behavioural flexibility, motor inhibition, attention towards owner, instruction following, delay inhibition and working memory. These factors appear similar to factors identified in human EF scales. Working dogs exhibit higher EF scores on the DEFS than non-working dogs. Dogs sourced from breeders exhibited higher DEFS scores than dogs sourced from shelters, and the amount of training received positively correlated with dogs’ DEFS scores. The DEFS requires further validation with cognitive measures. The DEFS could then be used by researchers to complement assessment of dogs’ EF skills through cognitive measures or assess dogs’ EF skills in large samples.

**Supplementary Information:**

The online version contains supplementary material available at 10.1007/s10071-022-01629-1.

## Introduction

Executive functions (EFs) are cognitive processes that are used to effortfully self-regulate behaviour (Diamond 2013; Pecora et al. 2017). EFs comprise components, such as inhibition, working memory, and cognitive flexibility. These abilities are required to focus on stimuli in the presence of distractions, to briefly keep useful information accessible in memory, and to flexibly alter behaviour due to changes in the environment (Pecora et al. 2017). EFs are needed to control behaviour in a deliberate way, instead of reacting to stimuli as they appear.

Being able to control behaviour deliberately appears to be crucial for dogs’ success in the current human-dominated environment. Dogs often depend on humans as their caregivers. Two such situations are dogs living as pets in people’s homes, and dogs taking up working roles that assist humans in various tasks (Bray et al. 2017; Cobb et al. 2015; Cohen 2018; Rooney et al. 2009; Troisi et al. 2019). It appears that effortful self-regulation of behaviour is crucial for dogs in these situations. For example, pet dogs must inhibit the impulse to chew on their owners’ shoes; guide dogs must ignore distractions while leading their handler; and scent detection dogs must keep a target scent in mind while searching the environment.

A growing body of research is aiming to measure EF skills in dogs using cognitive measures (Horschler et al. 2019; Olsen 2018). This body of research remains limited in some respects (e.g. small sample sizes, lacking reliability measures, Olsen 2018). Nevertheless, it appears that EF skills can successfully be assessed through cognitive measures in dogs and early studies show that EF skills appear to be related to life outcomes such as working dog success (Brady et al. 2018a; Bray et al. 2014, 2020b, 2021; Cohen 2018; Hare and Ferrans 2021; Kelly et al. 2019; Lazarowski et al. 2019a, 2019b; Lit 2009; MacLean and Hare 2018; Tiira et al. 2020; Troisi et al. 2019).

Cognitive measures are an invaluable tool to assess EF, leading to objective measures of cognitive traits. However, cognitive measures also pose some problematic features. For instance, cognitive measures have been known to fall victim to the task-impurity problem. The task-impurity problem describes the fact that the task used might rely on a variety of cognitive skills, not only the EF skill being examined. Additionally, many EF tasks rely on being novel to the subject, but the extent of novelty might vary from subject to subject (Gerst et al. 2017). Furthermore, testing a large number of subjects can be logistically challenging and time-consuming, leading to rather small sample sizes (Olsen 2018). An additional method to analyse dogs’ EF skills to complement cognitive measures is needed.

In humans, one ubiquitously used method to assess EF skills is through behavioural rating scales, either self-reported or reported by caregivers, such as parents or teachers (Holst and Thorell 2018; Isquith et al. 2013; Sherman and Brooks 2010; Thorell and Nyberg 2008). While scales are by their very nature a subjective tool to assess EF, they do come with a set of advantages. One advantage of scales that have been experimentally validated is that data collection is less time-consuming for the researcher, making it easier to assess EF in large samples. Additionally, a comprehensive suite of components of EF skills can be assessed simultaneously, without the need for administration of multiple cognitive tests. This means EF structure and relations of different EF components can be measured. Rating scales also aim to assess multiple EF skills in a variety of different situations, over an extended period, whilst removing the artificial environment inherent in laboratory-based cognitive assessments. In humans, behavioural rating scales have been shown to reflect expected scores of EF skills in clinical settings, are associated with biological markers and correlate with real-world function (Gerst et al. 2017; Isquith et al. 2013). Overall, these features of rating scales complement the assessment of EF skills through cognitive measures.

Dog behaviour can be rated by their owners and caregivers. This method has been successfully used to measure traits, such as personality (Ley et al. 2009), aggression (van den Berg et al. 2010), impulsivity (Wright et al. 2011) and Attention deficit hyperactivity disorder-like traits (Vas et al. 2007). Aggression, impulsivity and ADHD are related to EF skills in humans (Gathercole et al. 2008; Kimonis et al. 2006), and this might be true in dogs also. Development of a scale to assess EF in dogs therefore appears feasible. Furthermore, traits measured in rating scales can be linked to dogs’ performance in cognitive measures (Brady et al. 2018b; Bunford et al. 2019; Wright et al. 2012). This shows the potential merit of assessing dog EF skills via owner ratings. To date, however, there is no comprehensive scale to assess a suite of EF skills in a healthy population of dogs.

The aim of this study is the development of a scale that can assess behavioural manifestations of dogs’ EF skills through owner ratings. The first research question was whether there are differences in how owners rate dog behaviours that might relate to the dog’s EF capacities. If yes, the further question was to determine whether distinct components relating to different EF skills, for example working memory or inhibition, could be identified.

## Methods

A survey with items aimed at measuring EF skills belonging to various EF components, such as inhibition, working memory and cognitive flexibility, was distributed online to a large convenience sample of dog owners. Exploratory and confirmatory factor analysis was used to identify and confirm different EF components. To assess ecological validity of the scale, dogs’ scores on the scale were compared across demographics, such as working status (working/non-working), source of acquisition (breeder/shelter) and amount of training received. Scale development steps followed practices described by DeVellis (2016). This study was approved by the La Trobe University Science, Health and Engineering College Human Ethics Sub-Committee, (approval number: HEC19533).

### Survey development

#### Item generation

A pool of items was generated, aiming to capture dog behaviours potentially related to EF via owner reports. Items were generated by adapting existing dog behaviour rating scales (Bray et al. 2017; Hsu and Serpell 2003; Wright et al. 2011) and scales aimed at measuring EF and related concepts in humans (Biederman et al. 2008; Roberts et al. 2011; Vallat-Azouvi et al. 2012). Some items were newly generated by the first author. The item pool was discussed with colleagues and modified accordingly. In total, 83 items were retained at this point.

#### Focus groups

In February and March 2020, two focus groups were held with people knowledgeable about dog behaviour, including veterinarians, dog trainers, kennel attendants, and other dog behaviour professionals. The first focus group, with seven participants, was held in person at La Trobe University’s Bundoora campus in Melbourne, Australia. Invitations for the first focus group were distributed via e-mail to local dog schools, dog rescue groups, veterinarians, and pet shops, as well as social media. A second focus group, with five participants, was held via Zoom videoconference software, due to COVID-19 gathering restrictions, with staff from a guide dog breeding and training organisation. Invitations for the second focus group were distributed via the organisation’s management to staff that closely work with the dogs.

Focus groups were chosen to cover a broad variety of people having good knowledge of dog behavioural cues through their work. After the first focus group was held, the opportunity arose to work with staff from a guide dog breeding and training organisation. As this group was not represented in the first focus group, a second focus group was held. The same dog behaviours were emphasised during both focus groups.

In both focus groups, EF and its role in behavioural self-regulation were explained to participants. Then, there was a discussion about potential observable dog behaviours and traits related to attention, WM, inhibition, and cognitive flexibility, resulting in new item generation. After this discussion, participants were provided with the list of previously generated items. These were discussed and modified. Both focus groups were audio recorded. Results from the focus group were discussed among the authors, and a total of 65 items were retained (Supplementary Material, Table 1).

### Survey administration

The generated items were used to create an online survey, which also included demographic questions about the participant (i.e., gender, age, country of birth) and the participant’s dog (i.e., age, sex, source of acquisition, training history). Participants had to take care of at least one dog and be at least 18 years of age. Invitations to take part in the study were shared worldwide via social media. Survey items (e.g., My dog often forgets what he/she was doing after getting distracted) were administered with a 5-point Likert scale ranging from “Never or almost never” to “Always or almost always”, with a “not applicable” option where necessary.

### Analysis

Item responses were reverse coded if needed, so that high numbers indicated high EF capabilities and low numbers indicated low EF capabilities. Only responses of participants that provided age data for the dog were retained. Participants with more than 10% missing responses were deleted. The obtained sample was split into subsamples by age of the dog (puppies: < 1 year, adults: 1–8 years, seniors: > 8 years). Only the adult sample was used for this study.

The adult sample was split in half randomly, with one subset used for exploratory factor analysis (EFA), as this extraction method is suited better for the development of scales than principal component analysis (Worthington and Whittaker 2006). The other subset was used for confirmatory factor analysis (CFA). Data cleaning was done using R version 4.0.0, and analysis was done using R version 4.0.0 and SPSS. Items with more than 15% missing responses were excluded, as they may have been difficult to answer or poorly worded. Items with extreme means (< 1.5 or > 4.5) were excluded.

#### Exploratory and confirmatory factor analysis

Likert scale surveys produce ordinal data. It is common to obtain factor solutions using correlation matrices that technically require continuous data (e.g., dimension reduction using principal axis factoring). However, polychoric correlations are suited for ordinal data and have been shown to produce more accurate results (Holgado-Tello et al. 2010).

Parallel analysis was performed in R using the function “fa.parallel” from the “psych” package. The parallel analysis results and the scree plot were analysed to determine the number of factors to be retained. The polychoric factor analysis was performed in R using the “fa” function from the “psych” package, with correlation method set to “poly” and pairwise deletion of missing values. Correlation of factors was analysed to determine whether an orthogonal or oblique rotation method was suitable. Confirmatory factor analysis was performed in R using the function “cfa” from the package “lavaan” with pairwise deletion of missing values and polychoric correlations.

For comparison, we repeated the exploratory factor analysis on the final factor solution obtained, using the more widespread approach of principal axis factoring with pairwise deletion of missing values, in SPSS. Factorability of the correlation matrix was determined using Barlett’s test of sphericity and the Kaiser–Meyer–Olkin test for sample adequacy (Worthington and Whittaker 2006). In both options, items were assigned to a factor if they loaded at |.3| or higher onto a single factor.

#### Correlations and group comparisons of demographics and subscale scores

Subscale scores were calculated for each participant by summing the scores for each item on each component and then dividing this number by the number of items. Correlations between subscale scores were calculated using Pearson’s correlations.

The scale scores were used for group comparisons with demographic factors. Categorical demographic data with two groups (i.e., owner gender, dog source, working dog status) were compared using t tests. Effect sizes were calculated using Cohen’s *d*, where 0.2 is typically considered a small, 0.5 a medium and 0.8 a large effect size (Cohen 1992). Categorical demographic data with more than two groups (i.e., dog sex/reproductive status) were compared using ANOVAs.

The relationships between subscale scores and training score were analysed using Pearson’s correlations. The training score was calculated by summing the different types of training the dog had received (e.g., puppy school, basic obedience at home, basic obedience in a dog school, agility, advanced obedience at home, scent training). The maximum score possible was 12. Due to the highly variable nature of individual training a dog received, quantifying the training a dog has received is a difficult task. This method of summarising all types of training a dog has received to create a numeric score is one way of making the training history of a dog accessible for analysis. A similar method of scoring training history has been used in previous studies (Chapagain et al. 2017; Wallis et al. 2014).

To avoid Type I errors with multiple tests on the same dependent variables (i.e., the scale and subscale scores) Bonferroni corrections were used. For easier interpretation of *p* values, the alpha level was kept at 0.05, and *p* values themselves were multiplied by 6, the total number of statistical tests per dependent variable.

## Results

### Participant demographics

A total of 1239 participants took part and 1066 of those provided age data for their dog. Of these, 755 responses belonged to dogs 1–8 years of age and were analysed in this study. Participants with more than 10% missing item responses were deleted. This left 714 participants aged 18–76 years (*M* = 37.81 years, SD = 12.71 years) of whom 87.8% were female (*n* = 627). Most participants were born in Australia (41.0%), followed by the USA (16.1%) and the UK (13.9%), with a small proportion of responses coming from various other countries. The sample was randomly split into two subsamples of 357 participants for the purpose of exploratory factor analysis and confirmatory factor analysis, respectively.

### Exploratory factor analysis

Two of the 65 items were excluded, as they had more than 15% missing responses and were likely difficult to answer or unclear. Both items were about warning signs for aggressive behaviour. Seven items with extreme means (< 1.5 or > 4.5) were excluded. A parallel analysis on the remaining 56 items from the first sample of 357 responses suggested 10 factors to be retained, while a visually identified inflection point in the scree plot suggested to retain six factors. Parallel analysis for EFA, an adjusted method of parallel analysis for PCA, is prone to overestimation of the factors to be retained (DeVellis 2016). An initial polychoric EFA with oblimin rotation on the remaining 56 items found six factors accounting for 36.3% of the common variance. Some factors were correlated with other factors, with correlations as high as 0.38; therefore, the oblique oblimin rotation method was used throughout.

In this exploratory study, an iterative procedure to gradually reach a final solution was employed. Multiple EFAs were computed, varying the number of factors from six to 10. Items that did not load onto a factor, and items that cross-loaded or were redundant within the factor, were excluded after each round of analysis. The final solution consisted of six factors and 23 items (Table 1), explaining 49.7% of the common variance. The degrees of freedom for the model are 130. The factors were conceptually distinct and concise, each consisting of three to four items loading at or above 0.44 on a single factor (Table 2). Cronbach’s Alphas were computed to determine internal consistency of the factors; all six factors reached reliability scores higher than 0.60 (Table 1), which is an acceptable score for reliability in exploratory research (Litwin and Fink 2003).

**Table 1:** Polychoric exploratory factor analysis with the items retained in final solution

	Label	Item	Factor					
			1	2	3	4	5	6
Behavioural flexibility	BF1	My dog gets upset about changes in the environment (e.g. a new piece of furniture)	**0.51**	− 0.03	− 0.02	− 0.05	0.03	0.10
	BF2	My dog can relax in public places (e.g. a café)	**0.69**	0.21	0.01	− 0.02	0.05	− 0.01
	BF3	My dog adapts well to new situations and environments	**0.88**	− 0.12	0.03	0.00	− 0.01	0.04
	BF4	My dog can relax in unfamiliar environments (e.g. a friend’s house, a holiday home)	**0.77**	0.11	0.00	0.04	0.04	− 0.03
Motor regulation	Mot_Reg1	My dog gets excited around other dogs	0.01	**0.57**	0.09	− 0.01	− 0.09	0.00
	Mot_Reg2	My dog gets over-excited about things and can be a bit "over the top" at these times	0.11	**0.75**	0.09	− 0.02	0.01	0.04
	Mot_Reg3	Overall, my dog is excitable	− 0.04	**0.92**	− 0.03	− 0.02	0.02	− 0.02
	Mot_Reg4	My dog needs constant reminding to control behaviours which are inappropriate (e.g. jumping up on visitors)	0.09	**0.44**	− 0.07	0.24	0.07	0.23
Attention towards owner	Att_Own1	I can easily get my dog's attention	0.07	0.02	**0.87**	0.00	− 0.05	− 0.01
	Att_Own2	I can hold my dog’s attention for minutes at a time	0.06	0.00	**0.66**	0.01	0.07	0.19
	Att_Own3	My dog gazes at me or turns toward me when I speak to him/her	− 0.07	0.03	**0.78**	0.11	0.07	− 0.08
Instruction following	Instruct1	My dog can follow an instruction for a minute (e.g. ‘sit’ or’stay’)	− 0.05	− 0.03	0.09	**0.71**	0.15	0.11
	Instruct2	My dog can follow an instruction (e.g. ‘stay’) in a quiet place (e.g. at home)	− 0.01	0.01	0.03	**0.83**	− 0.06	0.01
	Instruct3	My dog will follow instructions (e.g. 'sit' or 'stay') when the cue is slightly different than normal (e.g. change in tone or pitch)	0.20	0.05	0.14	**0.53**	0.01	0.09
	Instruct4	My dog will follow instructions (e.g. 'sit' or 'stay') given by a stranger	0.29	− 0.09	0.05	**0.44**	− 0.01	− 0.12
Delay inhibition	Del_Inh1	My dog finds it difficult to tolerate waiting for a reward	0.04	0.02	− 0.02	0.11	**0.68**	0.02
	Del_Inh2	My dog finds it difficult to tolerate waiting for a walk	0.05	0.16	− 0.03	0.17	**0.51**	− 0.17
	Del_Inh3	My dog finds it difficult to tolerate waiting for dinner	− 0.02	− 0.01	− 0.01	0.04	**0.71**	− 0.02
	Del_Inh4	My dog gets frustrated when he/she is not immediately rewarded for a behaviour	0.07	− 0.04	0.12	− 0.26	**0.65**	0.12
Working memory	WM1	When playing, my dog easily gets distracted by other things	− 0.02	0.00	0.19	0.08	0.03	**0.51**
	WM2	It is difficult for my dog to concentrate on a single activity (e.g. chewing, playing)	0.03	0.05	0.20	− 0.10	− 0.05	**0.60**
	WM3	My dog often forgets what he/she was doing after getting distracted (e.g. forgets about a toy or treat if a loud noise distracted him/her for a moment)	0.04	0.05	− 0.11	0.12	0.08	**0.68**
	WM4	My dog forgets about something he/she wanted once it is out of sight (e.g. toy, food)	0.06	− 0.04	− 0.07	0.14	− 0.03	**0.47**
		Eigenvalues	6.52	2.25	1.74	1.68	1.53	1.37
		Proportion of common variance (%)	10.0	8.9	8.5	8.2	7.5	6.5
		Cumulative common variance (%)	10.0	18.9	27.4	35.6	43.1	49.7
		Cronbach’s alpha	0.79	0.77	0.77	0.73	0.69	0.66
		Mean scores^a^ *N* = 714	3.85	2.82	4.25	3.87	3.59	3.55
		SD (*N* = 714)	0.88	0.88	0.66	0.76	0.84	0.67

Bold values represent the items loading onto the relevant factor

^a^Scale scores were calculated by summing the scores for each item on each component, and then dividing by the number of items. The scale for item ratings ranged from 1 to 5

**Table 2:** Standardised and unstandardised estimates of items for the 6-factor solution allowing covariance among latent factors

Latent variable	Indicator	*B* (unstandardised estimates)	*β* (standardised estimates)	SE	*Z*
BF	BF1	1.000	0.451	0.057	7.893
BF	BF2	1.894	0.855	0.029	29.031
BF	BF3	1.380	0.623	0.039	16.063
BF	BF4	2.008	0.906	0.029	31.215
Mot_Reg	Mot_Reg1	1.000	0.568	0.041	13.820
Mot_Reg	Mot_Reg2	1.573	0.893	0.026	34.907
Mot_Reg	Mot_Reg3	1.378	0.783	0.030	26.115
Mot_Reg	Mot_Reg4	1.203	0.683	0.043	15.976
Att_Own	Att_Own1	1.000	0.847	0.029	29.408
Att_Own	Att_Own2	1.038	0.879	0.031	28.680
Att_Own	Att_Own3	0.874	0.740	0.41	18.243
Instruct	Instruct1	1.000	0.855	0.036	23.841
Instruct	Instruct2	0.911	0.778	0.036	21.762
Instruct	Instruct3	0.808	0.690	0.045	15.502
Instruct	Instruct4	0.380	0.325	0.064	5.103
Del_Inh	Del_Inh1	1.000	0.867	0.034	25.482
Del_Inh	Del_Inh2	0.823	0.714	0.040	18.039
Del_Inh	Del_Inh3	0.742	0.644	0.040	16.178
Del_Inh	Del_Inh4	0.682	0.591	0.043	13.682
WM	WM1	1.000	0.754	0.040	18.614
WM	WM2	0.914	0.689	0.041	16.654
WM	WM3	0.782	0.589	0.043	13.605
WM	WM4	0.357	0.269	0.056	4.822

*p* values for all latent variables are < 0.001***

In SPSS, a six-factor solution on the remaining 23 items, using principal axis factoring, explained 60.62% of common variance, with all items loading at or above |.415| onto a single factor (Supplementary material). While the item loading values are slightly different, the items separate out into the same groupings as in the factor analysis using polychoric correlations, and a greater proportion of common variance (60.62%) is explained by the six factors. Factorability of the correlation matrix from the principal axis factoring analysis was confirmed using Barlett’s test of sphericity (approx. chi-square 2450.3, *df* = 253, *p* < 0.0001) and the Kaiser–Meyer–Olkin test for sample adequacy (0.823).

The factors were respectively named: Behavioural Flexibility (BF), Motor Regulation (Mot_Reg), Attention Towards Owner (Att_Own), Instruction Following (Instruct), Delay Inhibition (Del_Inh) and Working Memory (WM).

### Confirmatory factor analysis

A polychoric confirmatory factor analysis was conducted on the second sample of 357 responses, using the lavaan version 0.6-8 in R version 4.0.0, with pairwise deletion of missing values, allowing covariances between the latent variables and diagonally weighted least squares estimation. The degrees of freedom for the model are 215. To determine model fit of the solution, the model chi-square (*χ*^2^), the root mean square error of approximation (RSMEA), the comparative fit index (CFI), the Tucker–Lewis index (TLI) and the standardised root mean square residual (SRMR) are reported (Kline 2015). While the chi-square test rejected the model [*χ*^2^(215) = 501.802, *p* < 0.001], this is not surprising as the chi-square test is sensitive to sample size, with large sample sizes lowering the p value (Alavi et al. 2020). The RMSEA was 0.06, which is within the < 0.08 threshold for good fit. The CFI was 0.97 and the TLI was 0.96, both being above the ≥ 0.95 cut-off for excellent fit. The SRMR, with a value of 0.07, was within the < 0.08 threshold for good fit (Kline 2015). These fit indices indicate good model fit, suggesting that the items in our survey can be well modelled by a 6-factor solution. Furthermore, the model allowing covariances among the 6 latent factors, fits the data significantly better than an orthogonal model with 6 latent variables, (*χ*^2^(15) = 386.66, *p* < 0.001***) and significantly better than a model with a single latent factor for executive functions (*χ*^2^(15) = 704.49, *p* < 0.001***). Standardised and unstandardised regression coefficients for the six-factor solution allowing covariance between the factors are reported in Table 2.

### Correlations and group comparisons of demographics and subscale scores

#### Correlations between dog executive function subscales

Mean scores and standard deviations of subscales for the complete sample (*N* = 714) are provided in Table 1. The mean score for the overall scale is 3.66 (SD = 0.48, *N* = 714). All subscales are significantly positively correlated with each other (Table 3). However, most correlations have a small effect size (*r* = < 0.29). Four correlations have a medium effect size (*r* = 0.30–0.49), namely motor regulation and behavioural flexibility, attention towards owner and behavioural flexibility, attention towards owner and instruction following, and attention towards owner and working memory.

**Table 3:** Pearson correlation coefficients between EF subscales, *N* = 714

	Motor regulation	Attention towards owner	Instruction following	Delay inhibition	Working memory	
Behavioural flexibility	0.34***	0.30***	0.26***	0.29***	0.27***	
Motor regulation		0.25***	0.16***	0.23***	0.28***	
Attention towards owner			0.31***	0.18***	0.36***	
Instruction following				0.18***	0.29***	
Delay inhibition					0.14***	
Working memory						

Correlations with medium and large effect sizes are marked in bold

***p < 0.001

#### Differences in executive functions scores across owner and dog demographics

**Fig. 1:**
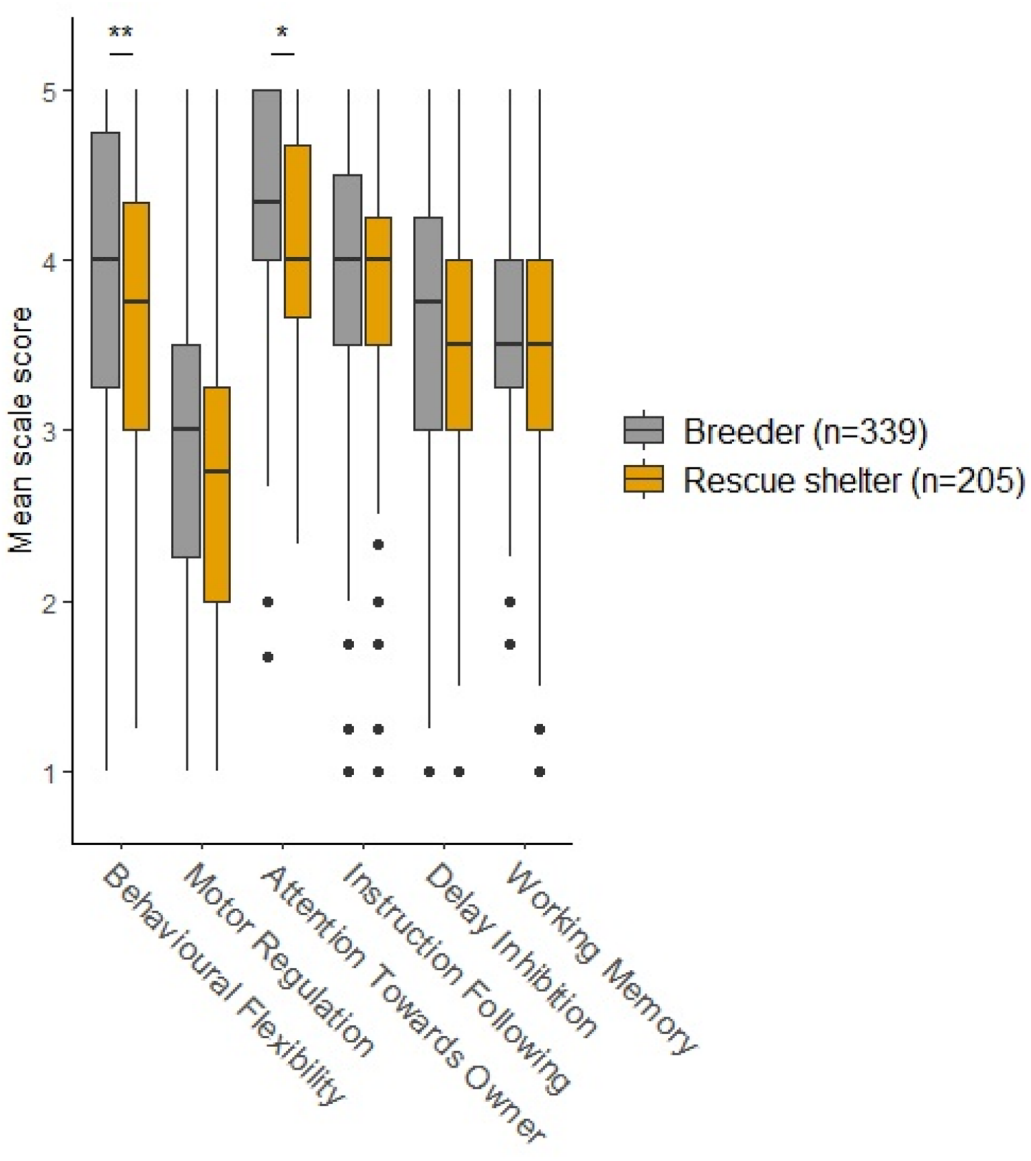
Dog source—subscale and total scale scores according to whether the dog was acquired from a rescue shelter (*n* = 205) or from a breeder (*n* = 339). *p* values have been Bonferroni-adjusted (multiplied by 5). Significance is indicated by *p ≤ 0.05, **p ≤ 0.01, ***p ≤ 0.001. Cohen’s *d* for behavioural flexibility was 0.31, for motor regulation was 0.23, for attention towards owner was 0.25 and 0.36 for the total scale, indicating small effect sizes

**Fig. 2:**
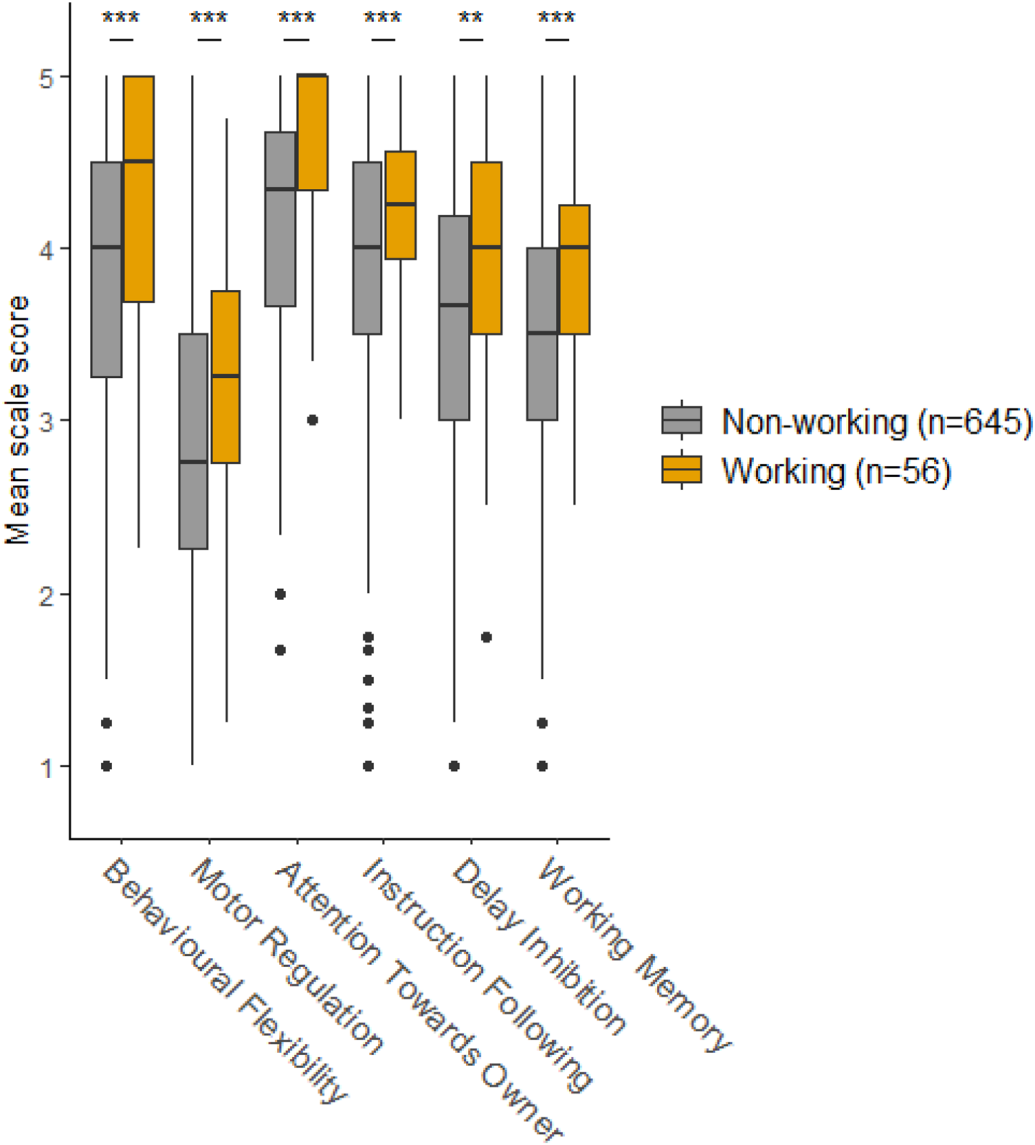
Working dog status—subscale and total scale scores according to whether the dog is a working dog (*n* = 56) or non-working dog (*n* = 645). *p* values have been Bonferroni-adjusted (multiplied by 5). Significance is indicated by *p ≤ 0.05, **p ≤ 0.01, ***p ≤ 0.001. Cohen’s *d* for: behavioural flexibility: 0.62; motor regulation: 0.52; attention towards owner: 0.68; instruction following: 0.61; delay inhibition: 0.49; working memory: 0.63.; total scale score: 0.94

Owner gender did not influence the dogs’ scores on any of the subscales (supplementary material). Dogs acquired from a breeder had significantly higher Behavioural Flexibility and Attention Towards Owner (see supplementary material for full descriptive and t test results) (Fig. 1), with small effect sizes. Working dogs had significantly higher scores than non-working dogs in all subscale scores with moderate effect sizes, except for Delay Inhibition (small effect size), Fig. 2 and supplementary material).

Two-way ANOVAs on the dogs’ sex (male or female) and reproductive status (desexed or intact) for the subscale scores indicate that female dogs had a significantly better motor regulation (*F*(1) = 11.09, *p* adjusted = 0.005) score than male dogs. Reproductive status was not significantly associated with any scale scores and no interaction effect was detected (supplementary material).

**Fig. 3:**
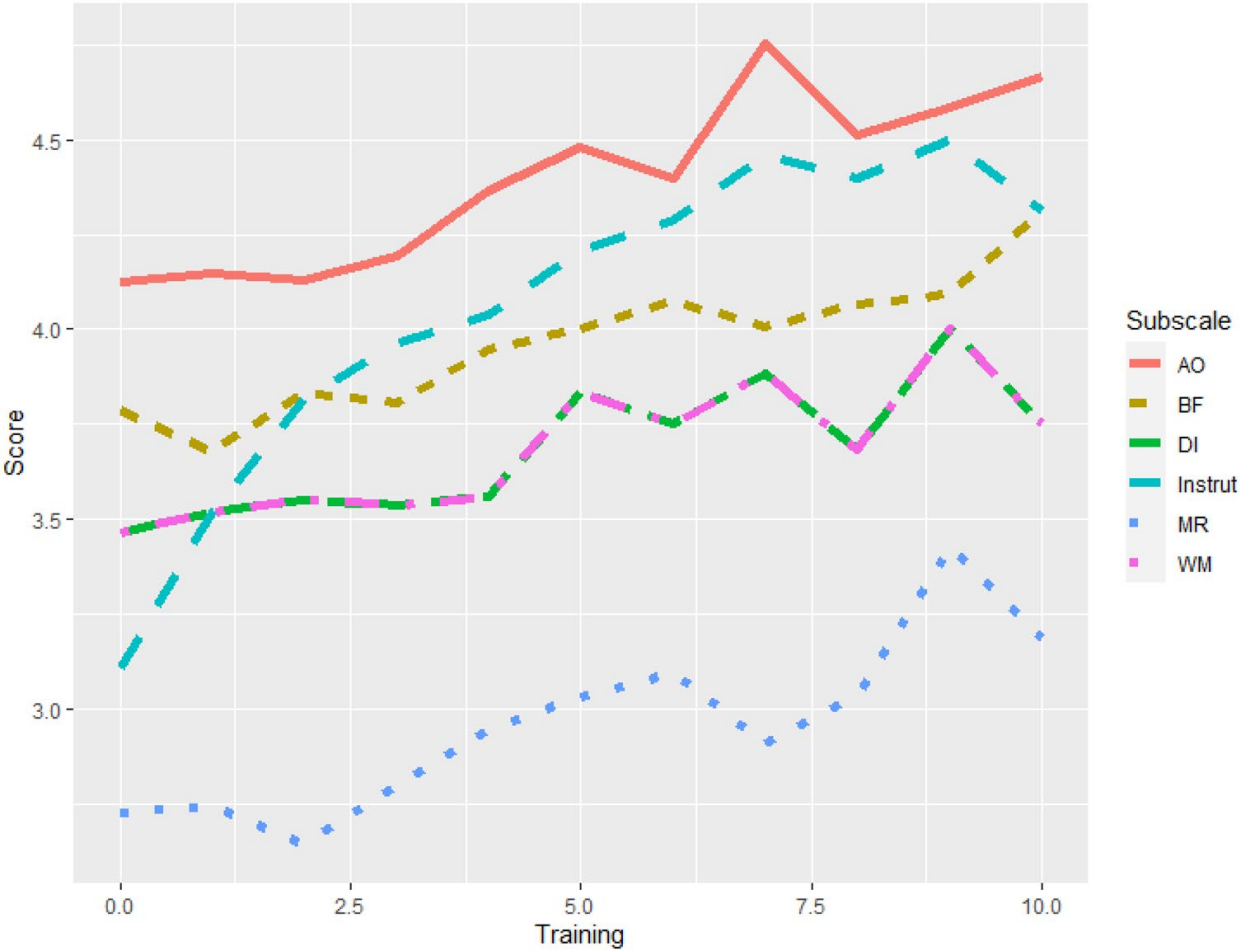
Overall executive function score by dogs’ training score. Training score was calculated by summing different types of training a dog had received

The dogs’ age was significantly positively correlated with Motor Regulation and Attention Towards Owner with small effect sizes (Table 4). The dogs’ training score, calculated by summing different types of training the dog had received throughout its life, was significantly positively correlated with all subscale scores. Most correlations had small effect sizes, but Instruction Following had a medium effect size (Table 4, Fig. 3).

**Table 4:** Pearson’s correlations of subscale scores with dogs’ training score

	*r*	*p* adjusted
Training score		
BF	**0.14**	0.001**
Mot_Reg	**0.15**	< 0.001***
Att_Own	**0.22**	< 0.001***
Instruct	**0.38**	< 0.001***
Del_Inh	**0.12**	0.01**
WM	**0.22**	< 0.001**
Dogs’ age		
BF	− 0.01	1.000
Mot_Reg	**0.21**	< 0.001***
Att_Own	**0.11**	0.020*
Instruct	0.01	1.000
Del_Inh	− 0.09	0.104
WM	0.07	0.356

*p* values have been Bonferroni-adjusted (multiplied by 6). Significance is indicated by *p ≤ 0.05, **p ≤ 0.01, ***p ≤ 0.001

## Discussion

This study aimed to start the development and validation of a dog executive function rating scale (DEFS) using behavioural ratings by owners. While assessment of dogs’ EF through cognitive measures has seen increased research interest over the past years (Olsen 2018) and behavioural rating scales are a feasible instrument to assess dogs’ traits (van den Berg et al. 2010; Wright et al. 2011), this is the first rating scale to measure multiple EF components in dogs. A preliminary list of 83 observable dog behaviours was revised with the help of two focus groups with participants highly knowledgeable in dog behaviour. The revised list of 65 observable dog behaviours was rated by 714 owners of dogs aged 1–8 years. Using half of the sample, data screening removed 9 items, and an iterative approach using polychoric exploratory factor analysis on the 56 items lead to 23 items remaining. For these 23 items, 49.7% of the common variance (or 60.62% of common variance using principal axis factoring) could be explained by 6 factors, containing three to four items each. Confirmatory factor analysis on the second half of the sample produced good model fit indices, suggesting that dogs’ EF can be well modelled by a six-factor solution.

### Interpretation of factor structure of dogs’ EF

The six factors of dogs’ EF identified in this study appear interpretable and can be partly matched to EF components previously identified. The first factor extracted, which we named behavioural flexibility, contains items describing the dog’s ability to adapt to new situations (Table 1). Previously, research into flexibility in dogs comes from experiments aiming to measure cognitive flexibility (CF). Research has described cognitive flexibility (CF) as the skill enabling individuals to adjust to different environmental demands and conditions flexibly (Diamond 2013). To date, CF in dogs has been measured using reversal learning tasks, particularly when looking at cognitive decline during aging (Chapagain et al. 2018; Milgram et al. 2005; Piotti et al. 2018). Behavioural flexibility, as measured in our scale, could be related to CF. When considering items describing the dog’s ability to be comfortable or relax in different settings as a measure of CF, in comparison to experimental tasks such as reversal learning, it must be considered that CF is likely a complex trait. While CF in humans is measured experimentally with tasks that require rapid mental switching such as the Wisconsin Card Sorting Task (Tchanturia et al. 2012), in scales it is measured through items such as “I get upset if other people disturb my plans for the day by being late” or “once I get into an emotional state, e.g. anger or sadness, it is difficult to soothe myself” (Roberts et al. 2011). It is likely that both aspects, the more isolated measure of CF in an artificial environment, and the broader measure of flexibility in applied everyday situations together can give us a better picture. However, the items in this factor could also be interpreted to reflect behaviours associated with neurotic personality traits in dogs (Ley et al. 2009). In humans, neuroticism has been found to be associated with cognitive flexibility (Clarke and Kiropoulos 2021; Zarei et al. 2018). While we are not aware of studies directly assessing the personality trait neuroticism and cognitive flexibility in dogs, it is possible that they are correlated in dogs as they are in humans. Future studies could aim to investigate this.

The second factor identified includes a form of regulation. The factor we called Motor Regulation contains items describing the dog’s ability to control motor functions in situations of high arousal (e.g. jumping up on visitors). Ability to control pre-potent motor responses has been assessed in dogs using tasks, such as the A-not-B task and cylinder tasks (Barrera et al. 2018; Bray et al. 2020a; Fagnani et al. 2016), which are classed as a form on inhibition. Research has described inhibition being used to control pre-potent behavioural responses, thoughts, and attention (Diamond 2013). The Motor Regulation factor might be measuring a similar construct. However, Motor Regulation appears to have similarities to behaviours previously measured as “excitability” also (Hsu and Serpell 2003). In humans, emotional regulation has been associated with inhibition (Leen-Feldner et al. 2004; Reese et al. 2015). Emotional regulation shares traits with excitability in dogs, as it includes the inhibition and modulation of emotions and accompanying behaviours (Eisenberg and Spinrad 2004). A link between excitability and inhibition might exist in dogs also.

The fifth factor identified in this study, Delay Inhibition, contains items describing the dog’s ability to control its behaviour when waiting for something highly anticipated (e.g. dinner, walks). Tasks that have been used to measure inhibition in dogs that feature a delay or waiting component for a reward are called delay discounting tasks (Riemer et al. 2014). In their study, Riemer et al. (2014) differentiated between motor and cognitive impulsivity, which might be analogues to our factors of motor regulation and Delay Inhibition. Our result of two separate factors pertaining to concepts that can be related to different forms of inhibition is consistent with research showing that inhibition is context-dependent in dogs (Bray et al. 2014), and might comprise dissociable cognitive skills.

The third factor extracted, which we named Attention Towards Owner, contains items describing the dog’s attention towards its owner. Attention is an underlying cognitive skill associated with EF (Garon et al. 2008); it is the ability to focus on one task or stimulus and is vital for any goal-directed behaviour. Studies examining attention in dogs have measured dogs’ eye contact towards moving objects or humans (Chapagain et al. 2017; Wallis et al. 2014). While attention towards the owner appears to be just a very small part of the dog’s possible attention repertoire, it is likely the easiest for owners to observe. Dogs likely differ in their selective and focused attention towards multi-sensory stimuli in the environment. However, it is likely that owners do not always pay attention to the dog, and behavioural ratings of attention towards the environment therefore did not emerge as a factor in our scale. Additionally, dogs originate from wolves, which show co-operative behaviours, and have been selected for mental adaptation for roles in the human society (Cooper et al. 2003). Ability to pay attention to the owner makes dogs exemplary models for social cognition (Kubinyi et al. 2007).

The fourth factor identified, named Instruction Following, consists of items describing how the dog follows instructions and cues given in different ways and situations. Instruction Following appears to be a higher-order EF component, which might draw upon skills, such as attention towards the instruction given, inhibition of distractions, and working memory to keep the instruction in mind during execution. Instruction Following does rely on the dog having been trained on the given instruction. Currently this is not specifically stated in the items pertaining to Instruction Following, even though it might be implied. However, any future refinements of the scale should consider amending these items to clearly specify this (i.e. “my dog can follow an instruction that is has been taught, for a minute”).

To be able to interpret this factor better, it might be prudent to change the items within it to include this caveat, e.g., “my dog can follow an instruction that it has been taught, for a minute”. While instruction following per se has not been examined as a cognitive skill in dogs, there might be similarities to measures of trainability (Hsu and Serpell 2003).

The last factor extracted in our study, which we termed Working Memory, contains items describing concentration on a task and keeping objects or activities in mind when no longer perceptually present. Previously, working memory has been defined as a cognitive skill enabling individuals to temporarily hold information in a state of increased accessibility (Cowan 2017). Cognitive tasks used in dogs to measure working memory typically include different versions of delayed response tasks (Bray et al. 2020a; Fiset et al. 2003; Krichbaum et al. 2021).

The three most commonly named basic components of EF in the literature are inhibition, working memory and cognitive flexibility (Diamond 2013; Garon et al. 2008; Pecora et al. 2017). All three have factors that might correspond in the dog EF factor structure described in this study, namely the factors motor regulation and delay inhibition, working memory and behavioural flexibility. Attention is another underlying cognitive skill associated with EF (Garon et al. 2008), which can be observed in dogs in the attention towards owner factor. As discussed previously, this is only a narrow part of the domain of attention towards the environment in dogs, but likely one that is relatively easy for owners to assess. It is likely influenced by experiences with the owner (e.g. training). Whether this part of attention is correlated to other measures of attention in dogs remains to be seen. The remaining factor, Instruction Following, is likely a higher-order EF component, drawing upon other EF skills, such as attention towards the instruction given, inhibition of distractions, and working memory to keep the instruction in mind during execution.

All subscale scores are significantly correlated with small to medium effect sizes (Table 3). Given the complexity of factors, genetic and environmental (Foraita et al. 2021), that are likely to influence dogs’ EF, this is not surprising. Indeed, different executive function components have been found to be moderately correlated in humans, but clearly separable (Miyake et al. 2000).

To our knowledge, the only description of factor structure of EF so far has been in humans. The number of factors described varies between studies. One commonly used scale to assess EF in humans is the Behaviour Rating Inventory of Executive Function (BRIEF), originally developed for school-aged children (BRIEF, Gioa et al. 2000), and adapted for adults (BRIEF-A, Roth et al. 2005) as well as pre-schoolers (BRIEF-P, Sherman and Brooks 2010). The number of factors ranges from five in pre-schoolers, to eight in school-aged children and nine in adults, coherent with the notion that EF might be a more unitary construct early in development, becoming more complex over time (Garon et al. 2008). Six EF factors identified in dogs falls in between five and eight different factors in preschool and school-aged children, respectively.

Many of the BRIEF factors appear similar to factors identified in our study. The factors in the BRIEF-P are inhibit, shift, emotional control, working memory and plan/organize (Sherman and Brooks 2010). Inhibit is described as impulse control and regulating of behaviour. This appears to be equivalent to our factor motor inhibition. Shift is described as the ability to switch between activities. This appears similar to our factor behavioural flexibility. Emotional control is the ability to modulate emotional responses, and might have similarities to our factor delay inhibition, which describes dogs’ ability to modulate their emotional response when waiting for an anticipated positive stimulus (e.g. dinner, walks). Emotional control might also be related to our factor motor inhibition, which describes dogs’ ability to control motor responses in exciting (i.e. emotional) situations. Working Memory in the BRIEF-P, described as the ability to hold information in mind and staying with an activity, appears equivalent to our factor working memory. The plan/organize scale of the BRIEF-P appears quite human specific, described as the ability to set goals and develop steps ahead of time to complete tasks, and does not match any of our factors. Two of our factors, attention towards owner and instruction following, appear to be dog-specific. Overall, there appears to be considerable overlap in the factor structure of EF in human children and dogs.

### Associations of scale scores and owner and dog traits

Environmental variables are thought to influence EF in dogs (Foraita et al. 2021). Among those potential influencing factors are working dog status, dog source as well as the amount of training received. These domains might encompass a broad spectrum of influences for the individual dog, e.g. working dogs come from various backgrounds and are trained for various tasks, dogs from a shelter might have experienced more or less severe hardships. However, on a population level, we can expect differences, e.g. on average working dogs received a more structured training than the pet dogs, on average, shelter dogs have experienced more stress than pet dogs. Therefore, comparing these groups can provide us some insight into differences in cognition.

Dog cognition differs between working dogs and non-working dogs. For instance, working dogs have been found to exhibit higher inhibitory control than non-working dogs (Barrera et al. 2018). Dog cognition has also been shown to differ between pet and shelter dogs, with pet dogs exhibiting better inhibitory control than shelter dogs (Fagnani et al. 2016). Training might enhance dogs’ EF development (Foraita et al. 2021). For example, amount of training received is associated with focused and selective attention in dogs (Chapagain et al. 2017). If the scale is capturing dogs’ EF skills adequately, differences in scale scores between dog populations with different EF skills should be detected.

Indeed, we found that working dogs, who are often specifically bred for their roles and undergo meticulous training, achieved higher scores in all subscales and the overall scale score than non-working dogs. Additionally, the amount of training dogs had received was positively correlated with all subscale scores. Training is one factor that is thought to affect development of EF in dogs (Foraita et al. 2021). Differences in EF scores were also detected between dogs sourced from breeders and dogs sourced from shelters, with breeder-sourced dogs achieving higher scores in Behavioural Flexibility than shelter-sourced dogs. Early experience, which is likely to be more favourable for dogs sourced from breeders, is a second factor that is likely to affect development of EF in dogs (Foraita et al. 2021). Overall, these results suggest that our scale might adequately capture dogs’ cognitive skills associated with EF.

### Conclusion

This study surveyed a large number of dog owners regarding their dogs’ cognitive skills, which may be associated with EF. The resulting scale requires experimental validation. It might then be used by researchers to either complement assessment of dogs’ EF skills through cognitive measures, or to easily assess dogs’ EF skills in large online samples, as perceived by the dogs’ owners. Due to the relatively small number of items making up each subscale the scale can be administered in a short amount of time. However, this also means the relevant constructs might be measured quite narrowly. To investigate the ecological validity of the scale, future research should aim to compare scale results with other cognitive measures, especially experimental cognitive tasks, and in various dog populations.

## Supplementary Information

Below is the link to the electronic supplementary material.

Supplementary file1 (DOCX 242 KB)

Supplementary file2 (PDF 102 KB)

Supplementary file3 (PDF 139 KB)

## Data Availability

No.
