# Supplementary material for: Development of the dog executive function scale (DEFS) for adult dogs
Source: Anim Cogn. 2022 May 17;25(6):1479–91. doi: 10.1007/s10071-022-01629-1 (PMC9113072; doi:10.1007/s10071-022-01629-1)
Supplement: Supplementary file 1 — Supplementary file1 (DOCX 242 KB) [file 10071_2022_1629_MOESM1_ESM.docx]

# Survey Items

Table 1 Items retained following the focus groups and used in the online survey. Items were presented in blocks in the survey. Block order was randomised across participants. Items were presented with a Likert scale from 1-5 (Never or almost never, Rarely, Sometimes, Often, Always or almost always), with a “not applicable” option where appropriate.

| Block 1 - Statements about how your dog controls behaviours towards himself, you, and others. |
| --- |
| My dog does not control his/her behaviour (e.g. jumps up on counter or people, when he/she should not). |
| My dog chokes/gags from eating too quickly. |
| My dog does not think before he/she acts (e.g. would steal food without first looking to see if someone is watching). |
| My dog can be very persistent. |
| My dog takes treats out of my hand gently (i.e. without hurting me). |
| My dog becomes frustrated when failing a task. |
| My dog shows warning signs (e.g. showing teeth, growling) before he/she snaps or lunges **at another dog.** |
| My dog shows warning signs (e.g. showing teeth, growling) before he/she snaps or lunges **at people.** |
|  |
| Block 2 - Statements about how changes in the environment or in your routine affect your dog. |
| My dog is creative in finding solutions when confronted with problems (e.g. a food puzzle). |
| My dog gets upset about changes in the environment (e.g. a new piece of furniture). |
| When a usual strategy does not work, my dog finds it difficult to change strategy (e.g. the usual path is blocked by a closed door, and the dog has to take a different path). |
| My dog adapts well to new situations and environments. |
| My dog gets upset if plans are changed last-minute (e.g. leash put on for walking, but then something comes up). |
| My dog comes when called **at home.** |
| My dog comes when called **in the park.** |
| My dog comes when called **while playing with another dog.** |
|  |
| Block 3 - Statements about how different things excite your dog. |
| When my dog gets excited it can lead to fixed repetitive behaviours (e.g. tail chasing/spinning in circles). |
| My dog can remain calm in exciting situations (e.g. visitors arriving). |
| My dog sometimes pees when he/she is excited. |
| My dog gets excited around other dogs. |
| My dog gets over-excited about things and can be a bit "over the top" at these times. |
| Overall, my dog is excitable. |
| My dog will **release a toy** when told to do so (e.g. when playing tug of war). |
| My dog will **release food** from its mouth when told to do so (e.g. when finding food on the ground). |
|  |
| Block 4 - Statements about how waiting for something or having to stop a behaviour impacts your dog. |
| My dog finds it difficult to tolerate waiting **for a reward**. |
| My dog finds it difficult to tolerate waiting **for a walk.** |
| My dog finds it difficult to tolerate waiting **for dinner.** |
| My dog gets frustrated when he/she is not immediately rewarded for a behaviour. |
| My dog finds it difficult to stop doing something (e.g. chasing another dogs ball) **when being told off by another dog.** |
| My dog finds it difficult to stop doing something **when I tell him/her to stop.** |
| Once my dog has started an activity, he/she finds it difficult to stop. |
| When I present a new toy to my dog, my dog engages with it for a long time. |
|  |
| Block 5 - Statements about how different things distract your dog. |
| Noises and/or smells can easily distract my dog. |
| When playing, my dog easily gets distracted by other things. |
| When on the leash, my dog lunges towards **other dogs.** |
| When on the leash, my dog lunges towards **people.** |
| When on the leash, my dog lunges towards **other animals (e.g. cats, birds).** |
| My dog walks on a loose lead. |
| It is difficult for my dog to concentrate on a single activity (e.g. chewing, playing). |
|  |
| Block 6 - Statements about how different situations affect your dog's focus, memory and calmness. |
| My dog often forgets what he/she was doing after getting distracted (e.g. forgets about a toy or treat if a loud noise distracted him/her for a moment). |
| My dog forgets about something he/she wanted once it is out of sight (e.g. toy, food). |
| My dog has trouble staying focused during training sessions if not reminded every few seconds. |
| My dog needs constant reminding to control behaviours which are inappropriate (e.g. jumping up on visitors). |
| My dog cannot stop barking once started. |
| My dog takes a long time to settle down enough to concentrate during training. |
| My dog remains calm at the vet or groomer. |
| My dog can relax in public places (e.g. a café). |
| My dog can relax in unfamiliar environments (e.g. a friend’s house, a holiday home). |
|  |
| Block 7 - Statements about how your dog follows instructions in different scenarios. |
| My dog easily follows simple instructions (e.g. ‘sit’). |
| My dog has difficulties following complicated instructions, such as ‘go to your place’, even if my dog has practiced them often. |
| My dog can follow an instruction for a minute (e.g. ‘sit’ or ’stay’). |
| My dog can follow an instruction (e.g. ‘stay’) in a **quiet place (e.g. at home).** |
| My dog can follow an instruction (e.g. ‘stay’) in a **busy environment (e.g. the park).** |
| My dog reacts to **subtle cues** (e.g. short, delicate hand-signals). |
| My dog reacts to **obvious cues** (e.g. loud voice cues, clear and visible hand signals). |
| My dog will follow instructions (e.g. 'sit' or 'stay') **when the cue is slightly different than normal** (e.g. change in tone or pitch). |
| My dog will follow instructions (e.g. 'sit' or 'stay') **given by a stranger.** |
|  |
| Block 8 - Statements about how your dog pays attention to his/her surroundings. |
| I can easily get my dog's attention. |
| I can hold my dog’s attention for minutes at a time. |
| My dog gazes at me or turns toward me when I speak to him/her. |
| My dog is easily bored during activities with me (e.g. pays attention to things other than me). |
| Squeaky toys or moving objects can easily capture my dog's attention. |
| Objects laying on the floor can easily capture my dog's attention. |
| My dog finds it hard to pay attention to his/her surroundings (e.g. walks into objects or people). |
| My dog is easily bored when playing (e.g. never plays with the same toy for more than a few minutes). |
|  |

# Exploratory Factor Analysis – Principal axis factoring in SPSS

Table 2 Exploratory factor analysis - principal axis factoring with direct oblimin rotation and pairwise deletion of missing cases in SPSS. N = 358

| Label | Factor 1 | Factor 2 | Factor 3 | Factor 4 | Factor 5 | Factor 6 |
| --- | --- | --- | --- | --- | --- | --- |
| BF3 | **-.808** | .096 | .047 | .029 | -.011 | .030 |
| BF4 | **-.747** | -.117 | .029 | .049 | .034 | -.050 |
| BF2 | **-.664** | -.212 | .018 | -.011 | .033 | -.020 |
| BF1 | **-.451** | .026 | -.027 | -.039 | .049 | .099 |
| Mot_Reg3 | .038 | **-.882** | -.046 | -.058 | .050 | -.019 |
| Mot_Reg2 | -.103 | **-.725** | .069 | -.030 | .043 | .031 |
| Mot_Reg1 | -.009 | **-.545** | .058 | -.014 | -.069 | -.013 |
| Mot_Reg4 | -.070 | **-.433** | -.058 | .196 | .102 | .232 |
| Att_Own1 | -.074 | -.029 | **.803** | .010 | -.053 | -.013 |
| Att_Own3 | .076 | -.037 | **.669** | .119 | .066 | -.046 |
| Att_Own2 | -.086 | -.010 | **.597** | .015 | .052 | .196 |
| Instruct2 | .065 | -.018 | .025 | **.757** | -.029 | .057 |
| Instruct1 | .113 | .005 | .084 | **.657** | .135 | .165 |
| Instruct3 | -.121 | -.081 | .129 | **.509** | .007 | .119 |
| Instruct4 | -.215 | .068 | .079 | **.415** | -.007 | -.077 |
| Del_Inh1 | -.031 | -.010 | -.007 | .078 | **.668** | .040 |
| Del_Inh3 | .017 | .028 | -.010 | .024 | **.654** | .003 |
| Del_Inh4 | -.069 | .068 | .102 | -.225 | **.630** | .084 |
| Del_Inh2 | -.036 | -.159 | -.026 | .143 | **.459** | -.137 |
| WM3 | -.050 | -.057 | -.092 | .080 | .073 | **.647** |
| WM2 | -.039 | -.047 | .164 | -.141 | -.027 | **.579** |
| WM1 | .022 | -.010 | .162 | .037 | .047 | **.507** |
| WM4 | -.046 | .024 | -.047 | .107 | -.026 | **.436** |
| Eigenvalues | 1.50 | 2.11 | 5.64 | 1.69 | 1.65 | 1.36 |
| Proportion of variance | 6.50 | 9.15 | 24.52 | 7.37 | 7.16 | 5.92 |
| Cumulatve variance | 6.50 | 15.65 | 40.18 | 47.54 | 54.70 | 60.62 |

# Confirmatory factor analysis – Path diagram


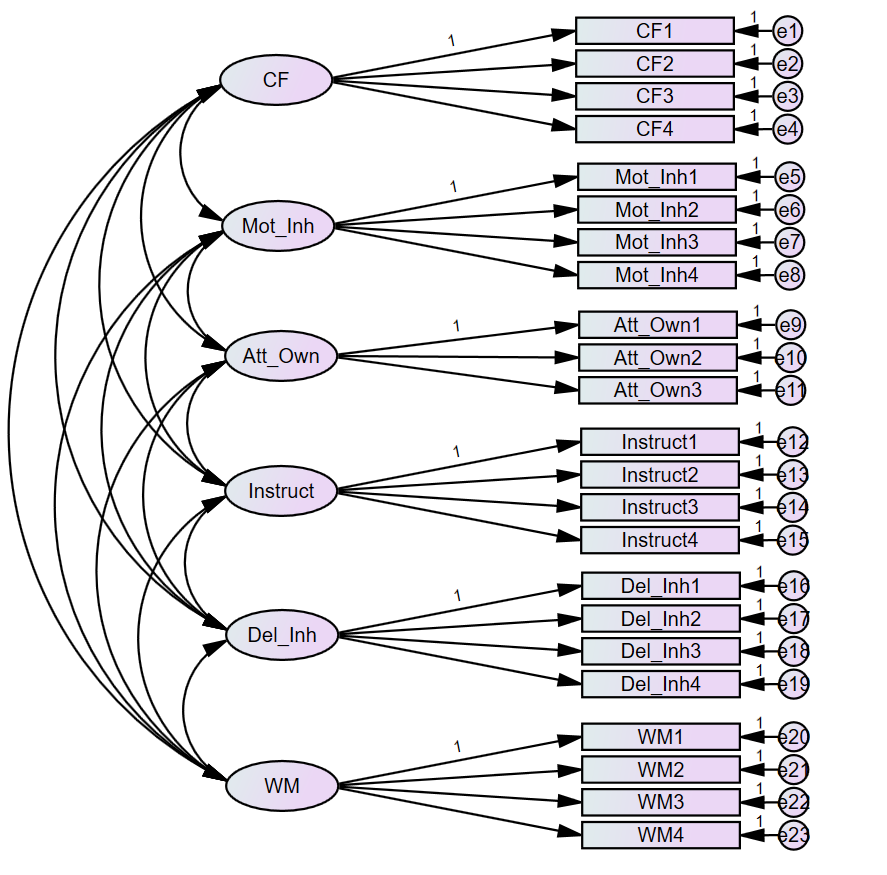


Figure 1 Path diagram of the 6-factor model with covariances among latent variables.

# Demographics

## Owner gender, working dog status & dog source

Table 3 Differences in subscale scores for categorical variables with two groups (Owner gender, working dog status, dog source)

| Owner gender | | | | | | | |  |
| --- | --- | --- | --- | --- | --- | --- | --- | --- |
|  | Female (n=626) | | Male (n=74) | | Welch two sample t-test | | |  |
|  | mean | SD | mean | SD | t | df | P adjusted | Cohen’s d |
| BF | 3.84 | 0.865 | 3.95 | 0.915 | -1.0679 | 89.127 | 1.000 | - |
| Mot Reg | 2.83 | 0.883 | 2.75 | 0.782 | 0.79051 | 96.424 | 1.000 | - |
| Att Own | 4.26 | 0.640 | 4.23 | 0.797 | 0.23326 | 84.476 | 1.000 | - |
| Instruct | 3.88 | 0.745 | 3.80 | 0.827 | 0.79221 | 87.555 | 1.000 | - |
| Del Inh | 3.60 | 0.835 | 3.52 | 0.878 | 0.74706 | 89.299 | 1.000 | - |
| WM | 3.54 | 0.662 | 3.70 | 0.780 | -1.7486 | 85.904 | 0.490 | - |
|  |  |  |  |  |  |  |  |  |
| Working dog status | | | | | | | |  |
|  | No (n=645) | | Yes (n=56) | | Welch two sample t-test | | |  |
|  | mean | SD | mean | SD | t | df | p adjusted | Cohen’s d |
| BF | **3.80** | **1.22** | **4.31** | **0.782** | **-4.6307** | **67.45** | **< .001***** | **0.62** |
| Mot_Reg | **2.78** | **0.884** | **3.20** | **0.738** | **-3.998** | **69.481** | **<.001**** | **0.52** |
| Att Own | **4.22** | **0.661** | **4.62** | **0.506** | **-5.5531** | **72.355** | **< .001***** | **0.68** |
| Instruct | **3.84** | **0.770** | **4.24** | **0.509** | **-5.3521** | **78.735** | **< .001***** | **0.61** |
| Del Inh | **3.56** | **0.844** | **3.95** | **0.762** | **-3.5886** | **67.264** | **.004**** | **0.49** |
| WM | **3.52** | **0.678** | **3.90** | **0.522** | **-5.1577** | **72.166** | **< .001***** | **0.63** |
|  |  |  |  |  |  |  |  |  |
| Dog source | | | | | | | |  |
|  | Breeder (n=339) | | Rescue shelter (n=205) | | Welch two sample t-test | | |  |
|  | mean | SD | mean | SD | t | df | p adjusted | Cohen’s d |
| BF | **3.94** | **0.859** | **3.67** | **0.890** | **3.5441** | **418.32** | **.003**** | **0.31** |
| Mot_Reg | 2.87 | 0.898 | 2.67 | 0.873 | 2.5969 | 439.92 | ..058 | 0.23 |
| Att Own | **4.30** | **0.664** | **4.14** | **0.639** | **2.6874** | **442.93** | **.045*** | **0.25** |
| Instruct | 3.93 | 0.725 | 3.82 | 0.714 | 1.7626 | 433.25 | .472 | - |
| Del Inh | 3.63 | 0.857 | 3.50 | 0.784 | 1.8825 | 460.06 | .362 | - |
| WM | 3.61 | 0.639 | 3.48 | 0.691 | 2.1522 | 404.64 | .192 | - |
|  |  |  |  |  |  |  |  |  |

## Dog sex and reproductive status

|  | Female/intact (n=52) | | Female/desexed (n=276) | | Male/desexed (n=296) | | Male/intact (n=90) | |  | Two-way ANOVA (unbalanced design) | | | | | |
| --- | --- | --- | --- | --- | --- | --- | --- | --- | --- | --- | --- | --- | --- | --- | --- |
|  |  |  |  |  |  |  |  |  |  | Sex | | Reproductive status | | Interaction | |
|  | mean | SD | mean | SD | mean | SD | mean | SD |  | F | p-adjusted | F | p-adjusted | F | p-adjusted |
| BF | 4.10 | 0.93 | 3.86 | 0.87 | 3.73 | 0.86 | 4.06 | 0.84 |  | 3.1495 | 0.458 | 3.4859 | 0.374 | 0.2678 | 1.000 |
| Mot Reg | 3.12 | 0.87 | 2.90 | 0.88 | 2.66 | 0.86 | 2.96 | 0.89 |  | **11.0888** | **0.005**** | 2.7738 | 0.577 | 0.2660 | 1.000 |
| Att Own | 4.41 | 0.65 | 4.27 | 0.64 | 4.20 | 0.68 | 4.25 | 0.65 |  | 1.4216 | 1.000 | 1.9773 | 0.961 | 0.5776 | 1.000 |
| Instruct | 3.87 | 0.74 | 3.90 | 0.74 | 3.87 | 0.73 | 3.78 | 0.91 |  | 0.1231 | 1.000 | 0.0671 | 1.000 | 0.1882 | 1.000 |
| Del Inh | 3.47 | 1.11 | 3.66 | 0.84 | 3.53 | 0.77 | 3.66 | 0.90 |  | 3.4030 | 0.393 | 2.3218 | 0.768 | 3.9756 | 0.280 |
| WM | 3.67 | 0.63 | 3.58 | 0.67 | 3.45 | 0.68 | 3.72 | 0.64 |  | 5.5887 | 0.110 | 0.8156 | 1.000 | 2.0120 | 0.939 |
