# Supplementary material for: Development of the dog executive function scale (DEFS) for adult dogs
Source: Anim Cogn. 2022 May 17;25(6):1479–91. doi: 10.1007/s10071-022-01629-1 (PMC9113072; doi:10.1007/s10071-022-01629-1)
Supplement: Supplementary file 2 — Supplementary file2 (PDF 102 KB) [file 10071_2022_1629_MOESM2_ESM.pdf]

Dog Executive Function Scale

Authors: Maike Foraita[1], Dr Tiffani Howell[1], Prof Pauleen Bennett[1]

1. Anthrozoology Research Group, School of Psychology and Public Health, La Trobe University, Melbourne, Australia

Please indicate to what extent the below statements apply to your dog. Some of these statements are very similar, so please read each one carefully. Please only tick 'not applicable' if you have never observed a situation like the one described in the statement. If you have more than one dog, please refer to the dog whose name starts with the letter closest to the letter 'A'.

Q		Never or almost never	Rarely	Sometimes	Often	Always or almost always	Not applicable
1	My dog gets upset about changes in the environment (e.g. a new piece of furniture).	☐	☐	☐	☐	☐	☐
2	My dog can relax in public places (e.g. a café).	☐	☐	☐	☐	☐	☐
3	My dog adapts well to new situations and environments.	☐	☐	☐	☐	☐	☐
4	My dog can relax in unfamiliar environments (e.g. a friend’s house, a holiday home).	☐	☐	☐	☐	☐	☐

		Never or almost never	Rarely	Sometimes	Often	Always or almost always	Not applicable
5	My dog gets excited around other dogs.	☐	☐	☐	☐	☐	☐
6	My dog gets over-excited about things and can be a bit "over the top" at these times.	☐	☐	☐	☐	☐	☐

7	Overall, my dog is excitable.	☐	☐	☐	☐	☐	☐
8	My dog needs constant reminding to control behaviours which are inappropriate (e.g. jumping up on visitors).	☐	☐	☐	☐	☐	☐

		Never or almost never	Rarely	Sometimes	Often	Always or almost always	Not applicable
9	I can easily get my dog's attention.	☐	☐	☐	☐	☐	☐
10	I can hold my dog’s attention for minutes at a time.	☐	☐	☐	☐	☐	☐
11	My dog gazes at me or turns toward me when I speak to him/her.	☐	☐	☐	☐	☐	☐

		Never or almost never	Rarely	Sometimes	Often	Always or almost always	Not applicable
12	My dog can follow an instruction for a minute (e.g. ‘sit’ or ’stay’).	☐	☐	☐	☐	☐	☐
13	My dog can follow an instruction (e.g. ‘stay’) in a quiet place (e.g. at home).	☐	☐	☐	☐	☐	☐
14	My dog will follow instructions (e.g. 'sit' or 'stay') when the cue is slightly different than normal (e.g. change in tone or pitch).	☐	☐	☐	☐	☐	☐
15	My dog will follow instructions (e.g. 'sit' or 'stay') given by a stranger.	☐	☐	☐	☐	☐	☐

		Never or almost never	Rarely	Sometimes	Often	Always or almost always	Not applicable
16	My dog finds it difficult to tolerate waiting for a reward.	☐	☐	☐	☐	☐	☐
17	My dog finds it difficult to tolerate waiting for a walk.	☐	☐	☐	☐	☐	☐
18	My dog finds it difficult to tolerate waiting for dinner.	☐	☐	☐	☐	☐	☐
19	My dog gets frustrated when he/she is not immediately rewarded for a behaviour.	☐	☐	☐	☐	☐	☐

		Never or almost never	Rarely	Sometimes	Often	Always or almost always	Not applicable
20	When playing, my dog easily gets distracted by other things.	☐	☐	☐	☐	☐	☐
21	It is difficult for my dog to concentrate on a single activity (e.g. chewing, playing).	☐	☐	☐	☐	☐	☐
22	My dog often forgets what he/she was doing after getting distracted (e.g. forgets about a toy or treat if a loud noise distracted him/her for a moment).	☐	☐	☐	☐	☐	☐
23	My dog forgets about something he/she wanted once it is out of sight (e.g. toy, food).	☐	☐	☐	☐	☐	☐
