# Supplementary material for: Development of the dog executive function scale (DEFS) for adult dogs
Source: Anim Cogn. 2022 May 17;25(6):1479–91. doi: 10.1007/s10071-022-01629-1 (PMC9113072; doi:10.1007/s10071-022-01629-1)
Supplement: Supplementary file 3 — Supplementary file3 (PDF 139 KB) [file 10071_2022_1629_MOESM3_ESM.pdf]

Dog Executive Function Scale – Coding Key

Authors: Maike Foraita[1], Dr Tiffani Howell[1], Prof Pauleen Bennett[1]

1. Anthrozoology Research Group, School of Psychology and Public Health, La Trobe University, Melbourne, Australia

Scores range from 1 to 5, with higher scores indicating higher executive function skills. Responses to items indicated in the table below need to be reverse coded.

**Normal coding**		**Reverse coding**	
Never or almost never	= 1	Never or almost never	= 5
Rarely	= 2	Rarely	= 4
Sometimes	= 3	Sometimes	= 3
Often	= 4	Often	= 2
Always or almost always	= 5	Always or almost always	= 1

Q		Reverse coding
1	My dog gets upset about changes in the environment (e.g. a new piece of furniture).	YES
2	My dog can relax in public places (e.g. a café).	
3	My dog adapts well to new situations and environments.	
4	My dog can relax in unfamiliar environments (e.g. a friend’s house, a holiday home).	
5	My dog gets excited around other dogs.	YES
6	My dog gets over-excited about things and can be a bit "over the top" at these times.	YES
7	Overall, my dog is excitable.	YES
8	My dog needs constant reminding to control behaviours which are inappropriate (e.g. jumping up on visitors).	YES
9	I can easily get my dog's attention.	
10	I can hold my dog’s attention for minutes at a time.	
11	My dog gazes at me or turns toward me when I speak to him/her.	

12	My dog can follow an instruction for a minute (e.g. ‘sit’ or ’stay’).	
13	My dog can follow an instruction (e.g. ‘stay’) in a quiet place (e.g. at home).	
14	My dog will follow instructions (e.g. 'sit' or 'stay') when the cue is slightly different than normal (e.g. change in tone or pitch).	
15	My dog will follow instructions (e.g. 'sit' or 'stay') given by a stranger.	
16	My dog finds it difficult to tolerate waiting for a reward.	YES
17	My dog finds it difficult to tolerate waiting for a walk.	YES
18	My dog finds it difficult to tolerate waiting for dinner.	YES
19	My dog gets frustrated when he/she is not immediately rewarded for a behaviour.	YES

20	When playing, my dog easily gets distracted by other things.	YES
21	It is difficult for my dog to concentrate on a single activity (e.g. chewing, playing).	YES
22	My dog often forgets what he/she was doing after getting distracted (e.g. forgets about a toy or treat if a loud noise distracted him/her for a moment).	YES
23	My dog forgets about something he/she wanted once it is out of sight (e.g. toy, food).	YES
